# Two Furanosesterterpenoids from the Sponge *Luffariella variabilis*

**DOI:** 10.3390/md15080249

**Published:** 2017-08-10

**Authors:** Peni Ahmadi, Masahiro Higashi, Nicole J. de Voogd, Junichi Tanaka

**Affiliations:** 1 Department of Chemistry, Biology and Marine Science, University of the Ryukyus, Nishihara, Okinawa 903-0213, Japan; peni.ahmadi@gmail.com (P.A.); higashi@sci.u-ryukyu.ac.jp (M.H.); 2 Naturalis Biodiversity Center, P.O. Box 9517, 2300 RA Leiden, The Netherlands; nicole.devoogd@naturalis.nl

**Keywords:** sesterterpenoid, sponge, cytotoxicity

## Abstract

Two new sesterterpenoids, **1** and **2**, were isolated from the sponge *Luffariella variabilis*. Their planar structures were characterized with spectroscopic analyses. The sole chiral center of compound **1** was elucidated as 12*R* by comparing observed and calculated optical rotation values. The configurations of compound **2** were determined by NMR and electronic circular dichroism (ECD) studies. Furthermore, compound **2** showed cytotoxicity at IC_50_ 1.0 µM against NBT-T2 cells.

## 1. Introduction

Among sessile marine macroorganisms, sponges have remained the richest sources of new bioactive molecules [1]. Those belonging to the family Thorectidae are known as sources of sesterterpenoids and other molecules [2,3,4,5,6,7,8,9,10,11,12]. The title sponge *Luffariella variabilis*, a representative species of this family, has been studied by several research groups and found to be prolific in secondary metabolites [2,3,4,7,11,12]. Of the metabolites, manoalide was originally reported as an antibiotic by Scheuer [2] from a Palauan specimen. It was found to have additional biological activity such as anti-inflammatory [3], inhibitory activity against phospholipase A2 [4] and NS3 helicase of hepatitis C virus [5], and has been distributed as a reagent for life science studies. Additional members of sesterterpenoids with a similar molecular scaffold have been reported from different collections of sponges of the same genus [6,7,8,9,10,11,12]. Here, we report structures of two new sesterterpenoids isolated from a specimen of *L. variabilis* collected at a deeper coral reef. 

## 2. Results and Discussion

### 2.1. Structure Elucidation

As a part of collaborative efforts to search for antiviral molecules [13], we constructed a small library of Okinawan marine invertebrates. Although the lipophilic extract of the title sponge did not inhibit the growth of target viruses, it showed moderate cytotoxicity with a unique ^1^H-NMR spectrum. It was then sequentially separated with silica gel columns to give compounds **1** and **2** as glassy or oily substances.

**Figure 1:**
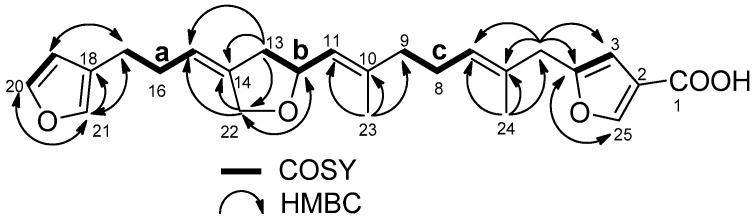
Substructures **a**–**c** and selected HMBC correlations of compound **1**.

The molecular formula of compound **1** was determined as C_25_H_30_O_5_ by observing its sodiated ion at *m*/*z* 433.19909 (∆ + 1.2 ppm). As eight out of eleven degrees of unsaturation can be accounted for by the presence of one carbonyl (1715 cm^−1^, δ_C_ 167.0 s) and seven double bonds (14 signals in the range from δ_C_ 156.4 to 106.0), the molecule was determined to contain three rings. Of the double bonds, two were incorporated into a β-substituted furan (δ_H_ 7.34 brs, 7.21 brs, 6.26 brs; δ_C_ 142.7 d, 138.9 d, 124.5 s, 110.9 d), while another two were assigned to a disubstituted furan moiety (δ_H_ 7.96 brs, 6.38 brs; δ_C_ 156.4 s, 147.7 d, 119.0 s, 106.0 d) after NMR inspection (Figures S1 and S2). Using 2D NMR (Figures S3–S5) analyses, three other double bonds were involved in the following three substructures: (**a**) –CH_2_–CH_2_–CH=C< (δ_H_ 5.33 m; δ_C_ 139.8 s, 119.2 d), (**b**) –CH_2_–CH(O–)–CH=C< (δ_H_ 5.24 m; δ_C_ 139.7 s, 124.9 d), and (**c**) –CH_2_–CH_2_–CH=C< (δ_H_ 5.24 m; δ_C_ 130.9 s, 127.2 d) (Figure 1). The remaining pieces of the molecule were an oxymethylene (δ_H_ 4.39 d, 4.20 d; δ_C_ 68.3), an sp^3^ hybridized methylene (δ_H_ 3.28 s; δ_C_ 38.1), and two vinyl methyls (δ_H_ 1.70 brs, 1.60 brs; δc_C_ 16.7, 15.9).

**Figure 2:**
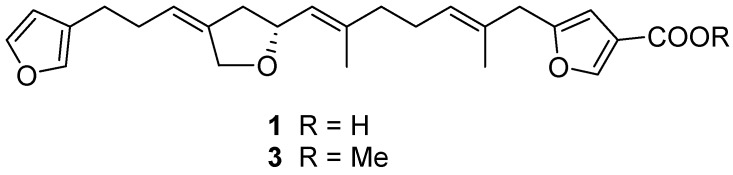
Structures of compounds **1** and **3**.

The oxymethylene group was incorporated into a five-membered ether ring connecting the substructures **a** and **b**, as shown by the HMBC cross peaks: H-22/C-12,14,15, H-12/C-22, and H-13/C-14,15,22. The sp^3^ hybridized methylene at δ_H_ 3.28 was flanked by the disubstituted furan and an olefin connected to substructure **c** by observing H-5/C-3,4,6,7,24 and H-24/C-5,6,7. Finally, additional correlations of H-23/C-9,10,11, H-17/C-19,21, and H-19,21/C-17 (Figure 1) allowed us to connect all of the above structural units except for the carbonyl group. The carbonyl group was at first judged as an ester from the chemical shift, however, it gave a methyl ester **3** (*m*/*z* 447.21457; δ_H_ 3.81 s; 1731 cm^−1^, Figure S6) on treatment with TMSCHN_2_, confirming it as a carboxylic acid. Both the ^1^H and ^13^C-NMR signals (δ_H_ 7.96; δ_C_ 147.7) for C-25 shifted more downfield than other furan α-positions such as C-21 (δ_H_ 7.21; δ_C_ 138.9), likely caused by conjugation with the carbonyl group. These chemical shifts are also comparable to those of β-carboxylic furans [14]. The double bond geometry at C-6 and C-10 was assigned as *E* by the chemical shifts of the vinyl methyls (δ_C_ 15.9 (C-24) and 16.7 (C-23)) and nuclear Overhauser effects (NOEs) (H-5/H-7, H-9/H-11, H-23/H-12), while the configuration at C-14 was determined as *Z* with an NOE (H-13/H-15). With all the NMR signals assigned, the planar structure was determined, as depicted in Figure 2 and Table 1.

**Figure 3:**
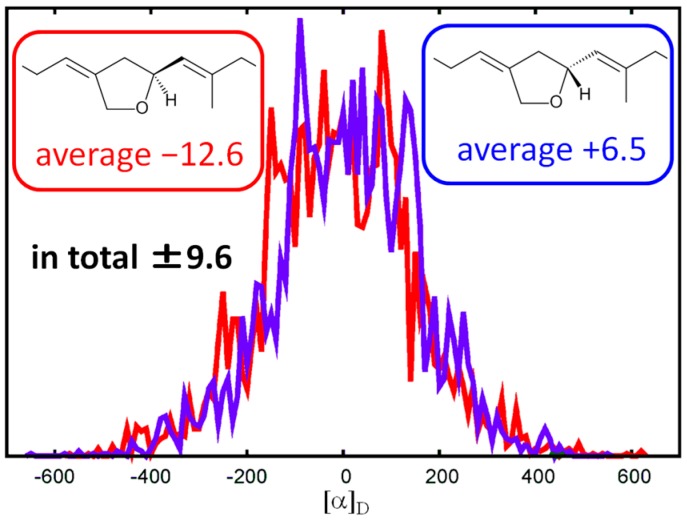
Distribution of calculated specific rotation values.

To elucidate the absolute configuration, we calculated the specific rotation values of (12*S*)-**1** and (12*R*)-**1** with molecular dynamics (MD) simulations and time-dependent density functional theory (TDDFT). After generating 5000 structures from a 500-ns MD trajectory for each chiral molecule in CH_2_Cl_2_ solutions, we calculated the specific rotation values with the TDDFT method. The resulting averaged optical rotation values of (12*S*)-**1** and (12*R*)-**1** were calculated as −9.6 and +9.6 (Figure 3). Since the natural **1** presented a specific rotation value of [α]_D_ +11, it is likely to have 12*R* configuration.

Compound **2** showed a sodiated ion at *m*/*z* 423.25046 (∆ − 1.6 ppm) in high resolution electrospray ionization mass spectrometry (HRESIMS), establishing the molecular formula C_25_H_36_O_4_ with eight degrees of unsaturation. As in compound **1**, a β-substituted furan moiety was deduced from the signals for α-positions at δ_H_ 7.35, 7.25; δ_C_ 142.6, 138.6 and for β-positions at δ_H_ 6.29; δ_C_ 125.4, 110.9 (Figures S7 and S8). The presence of an α,β-unsaturated δ-lactone moiety was inferred by observing an ester carbonyl (1705 cm^−1^; δ_H_ 166.5 s, C-1), conjugated olefinic signals (δ_H_ 6.63 brd; δ_C_ 128.2, 140.0, C-2,3), allylic methylene protons at δ_H_ 2.34 m and 2.21 ddd (H-4a,4b), and an oxymethine signal at δ_H_ 4.30; δ_C_ 81.9 (C-5). The lactone moiety was also confirmed by HMBC correlations (Figure S10) from a vinyl methyl at δ_H_ 1.92 s (H-25) to C-1,2,3 (δ_C_ 166.5, 128.2, 140.0) and from the vinyl proton H-3 to C-1,4,5,25 and COSY cross peaks (H-5/H-4a,4b and H-3/H-4a,25, Figure S11) and by a comparison of NMR data with those of schisanlactone B and colossolactone G [15,16]. The remaining two degrees of unsaturation are assignable to two additional rings.

**Figure 4:**
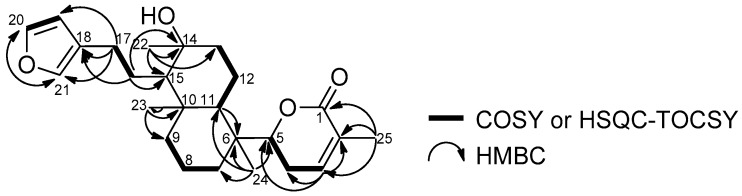
Selected 2D NMR correlations of compound **2**.

By analyzing the HMBC correlations from three singlet methyls at δ_H_ 1.18 (H-22), 1.03 (H-23), and 0.87 (H-24), it was straightforward to construct a partial structure starting from C-24 via C-6,7,11,10,9,15,14,13 to C-22. The two remaining methylenes at C-8 (δ_C_ 17.4) and C-12 (δ_C_ 17.5) were involved in the bicyclic portion by Heteronuclear Single Quantum Correlation-Total Correlation Spectroscopy (HSQC-TOCSY, Figure S12) correlations (H-7α C-8,9, H-11/C-12,13). Therefore, the decaline ring was confirmed. Two allylic methylene protons at δ_H_ 2.44 and 2.39 (H-17a,17b) were assigned next to the furan by observing HMBC correlations to furan carbons (H-17a,17b/C-18,19,21) and another methylene at C-16 (H-16/C-14,15,17,18). Since the δ-lactone moiety was confirmed to be attached to C-6 by observing an HMBC cross peak (H-24/C-5), the whole planar structure of compound **2** was determined, as depicted in Figure 4.

**Figure 5:**
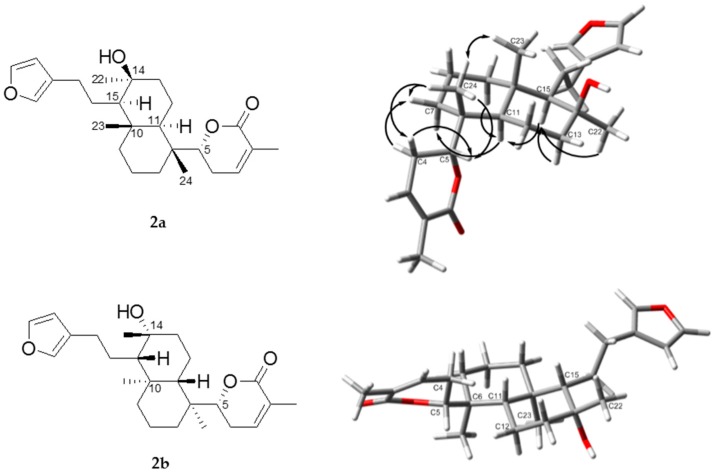
Structures of **2a** and **2b** and selected NOEs in arrows. The structures were optimized with the density functional theory (DFT) method at the IEFPCM (CHCl_3_)/B3LYP/6-31G(d) level. Red color means oxygen atom.

The absolute configuration of the δ-lactone was determined as *R* on the basis of its positive Cotton effect at 253 nm (∆ε + 27.6, Figure S13), which sign is the same as that of (+)-parasorbic acid with the same δ-lactone moiety [17]. The bicyclic portion takes the *trans*-decaline ring system with chair conformation by observing NOEs for H-15/H-11,22 and H-23/H-24 (Figure S14). Therefore, two candidate structures, **2a** and **2b**, are possible for the absolute configuration of compound **2**. In both cases, there is steric hindrance between the methylene protons at C-4 and at C-12. In fact, we could not observe any NOE between the methylene protons, confirming the restricted rotation between C-5/C-6. The DFT calculation also showed that one conformer is dominant with respect to the rotation between C-5/C-6 (Figure 5). The observed NOE between H-4b/H-24 and the absence of NOEs between H-4a,4b/H-11 support the structure **2a**, but not **2b**. In conclusion, the configurations of compound **2** were elucidated as 5*R*, 6*R*, 10*S*, 11*R*, 14*S*, and 15*R*. 

### 2.2. Biological Activity

Compounds **1**–**3** were tested against cultured NBT-T2 cells by MTT assay. Compounds **1** and **2** showed cytotoxicity at IC_50_ 47.5 and 1.0 µM, while compound **3** did not. 

## 3. Conclusions

Two new furanosesterterpenoids **1** and **2** were isolated from the Okinawan sponge *L. variabillis*, and their absolute stereochemistry was elucidated with spectroscopic analyses and calculation. Of the two molecules, compound **2** may be the first member with this skeleton. Specimens of *L. variabilis* collected at shallower coral reefs in Okinawa usually contain manaoalide as the major constituent, while the current specimen collected from a deeper reef at 60 m contained new sesterterpenoids without manoalide. We have previously encountered metabolite variation with depth [18], and the current work may be another example of this. 

## 4. Materials and Methods 

### 4.1. General Experimental Procedure

NMR spectra were recorded on a Bruker AVANCE III 500 spectrometer (Billerica, MA, USA). ESI mass spectra were taken on a Jeol JMS-T 100LP mass spectrometer (Tokyo, Japan). HPLC separation was done on a unit equipped with a Shimadzu LC-10AD pump (Kyoto, Japan), a Shimadzu SPD-10A UV detector, a Shodex RI-101 refractive index detector (Tokyo, Japan), and a Nacalai Cosmosil 5SL-II column (4.6 × 250 mm) (Kyoto, Japan). Specific rotation was observed on a Jasco P-1010 polarimeter (Tokyo, Japan). FTIR spectrum was measured on a Jasco FT/IR-300 spectrophotometer. ECD spectrum was obtained on a Jasco J-820 spectropolarimeter. 

### 4.2. Specimen

The specimen was collected by hand using a trimix rebreather at a depth of 60 m from a reef near Iriomote Island, Okinawa, in July 2013, and kept frozen until extraction. The specimen was identified as *Luffariella variabilis* by one of the authors (NJdV) and deposited at the Naturalis Biodiversity Center with the code RMNH POR 8677. 

### 4.3. Extraction and Isolation 

After thawing, the sponge (57 g, wet) was cut and macerated ca. 12 h in acetone (150 mL), three times. The acetone solution was filtered and concentrated under vacuum, and the residue was partitioned between EtOAc and H_2_O. The organic layer gave an extract (770 mg) after concentration. A portion (260 mg) of the extract was separated on a Merck silica gel 60 (63–200 µm) column using solvents stepwise, namely, *n*-hexane, *n*-hexane-EtOAc (9-1, 7-3, 1-1), EtOAc, EtOAc-MeOH (1-1), and MeOH, to give eight fractions. A portion (18.2 mg) of the seventh fraction (54.4 mg) was passed through a silica Sep-Pak^®^ 3 cc Vac cartridge (200 mg) with EtOAc to remove polar impurities, and its first fraction (14.6 mg) was subjected to HPLC (Cosmosil 5SL-II, *n*-hexane-EtOAc, 2-3) to give compound **1** (2.0 mg, 3.3% from the extract). A further amount of compound **1** was obtained with similar treatment. A portion (12.7 mg) of the fourth fraction was purified via HPLC (Cosmosil 5SL-II, *n*-hexane-EtOAc, 4-1) to give compound **2** (7.0 mg, 6.5%).

Compound **1**: Glass. ^1^H and ^13^C-NMR (CDCl_3_) see Table 1. HRESIMS *m*/*z* 433.19909, calcd. for C_25_H_30_O_5_Na 433.19854 (∆ + 1.2 ppm). FTIR (neat) 2923, 1715, 1695, 1549, 1295, 1198, 767 cm^−1^. [α] + 11 (*c* 0.14, CH_2_Cl_2_).

Compound **2**: Colorless oil. ^1^H and ^13^C-NMR (CDCl_3_) see Table 1. HRESIMS *m*/*z* 423.25046, calcd. for C_25_H_36_O_4_Na 423.25113 (∆ − 1.6 ppm). FTIR (neat) 3498, 2928, 1705, 1243, 1136, 781 cm^−1^. ECD (MeOH) nm (∆ε) 253 nm (+27.6). [α] + 68 (*c* 0.18, MeOH). UV (MeOH) 212 nm (logε 4.1).

### 4.4. Methylation of Compound ***1*** to ***3***

Eight drops of a solution of TMSCHN_2_ in hexane were added to compound **1** (0.2 mg) in MeOH (200 µL). After allowing the mixture to stand for 5 min at room temperature, it was concentrated with N_2_ flow to give 0.2 mg of the ester **3**.

Compound **3**: HRESIMS *m*/*z* 447.21457, calcd. for C_26_H_32_O_5_Na 447.21419 (∆ +0.8 ppm). FTIR (neat) 1731 cm^−1^. ^1^H-NMR (CDCl_3_) δ 7.88 (1H, s, H-25), 7.34 (1H, bs, H-20), 7.21 (1H, bs, H-21), 6.35 (1H, bs, H-3), 6.27 (1H, bs, H-19), 5.33 (1H, m, H-15), 5.23 (1H, m, H-7), 5.23 (1H, m, H-11), 4.55 (1H, dt, *J* = 5.8, 8.7 Hz, H-12), 4.38 (1H, d, *J* = 13.3 Hz, H-22a), 4.20 (1h, d, *J* = 13.3 Hz, H-22b), 3.81 (3H, s, OMe), 3.28 (2H, s, H-5), 2.58 (1H, dd, *J* = 5.5, 15.3 Hz, H-13a), 2.48 (2H, t, *J* = 7.4 Hz, H-17), 2.22 (1H, m, H-13b), 2.16 (2H, m, H-8), 2.16 (2H, m, H-16), 2.05 (2H, m, H-9), 1.70 (3H, s, H-23), 1.60 (3H, s, H-24).

### 4.5. Cytotoxicity Assay

NBT-T2 cells were incubated at 37 °C under 5% CO_2_ for 24 h in a 96-well plate containing 100 µL of Dulbecco’s Modified Eagle’s Medium (DMEM). An aliquot (1 µL) of DMSO solution of compounds **1**, **2**, and **3** was dispensed into each well in triplicate for each concentration and the cells were incubated for 48 h. Doxorubicin was used as a positive control, showing cytotoxicity at IC_50_ 0.5 µM. A 3-(4,5-dimethylthiazol-2-yl)-2,5-diphenyltetrazolium bromide (MTT) solution was prepared by dissolving 5 mg of MTT in 1 mL of phosphate-buffered saline (PBS), and the solution was added to 10 mL of DMEM. After removing the culture media and dispensing 100 µL of the MTT solution, the wells were incubated for additional 3 h. The solution was then removed and 100 µL of DMSO was added to each well. The absorbance of each well was measured at 570 nm using a microplate reader. IC_50_ values were obtained by analyzing the data on Kaleida Graph software (version 4.1, Synergy Software, Reading, PA, USA). 

### 4.6. Calculation of Specific Rotation Value

We first performed the MD simulation to sample various conformations of compound **1** in CH_2_Cl_2_ solution. The system consisted of one compound **1** and 3375 CH_2_Cl_2_ molecules. A cubic unit cell with the periodic boundary condition was employed, using a box length of 72.5 Å. The general Amber force field (GAFF) [19] was used for compound **1** and CH_2_Cl_2_. Long-range electrostatic interactions were treated with the particle mesh Ewald method. Bonds involving hydrogen were constrained by using the SHAKE method. The equations of motion were integrated using the leapfrog algorithm with a time step of 1 fs at a temperature of 300 K. The MD simulation was performed for 500 ns. Next, 5000 geometries of compound **1** taken from the MD trajectory were optimized with the DFT method at the IEFPCM(CH_2_Cl_2_)/B3LYP/6-31G(d) level. Finally, specific rotation values were calculated with the TDDFT method at the IEFPCM(CH_2_Cl_2_)/B3LYP/aug-cc-pVDZ level [20]. We performed this procedure for both 12*S* and 12*R* configurations. The distributions of specific rotation values for (12*S*)-**1** and (12*R*)-**1** were almost antisymmetric (Figure 1), indicating that the number of samples was sufficient. The specific rotation values averaged over 5000 samples were −12.6 and +6.5 for (12*S*)-**1** and (12*R*)-**1**, respectively. Further averaging the values over the two configurations, the values of (12*S*)-**1** and (12*R*)-**1** were −9.6 and +9.6, respectively. The MD simulations and TDDFT calculations were performed using the AMBER 12 [21] and Gaussian 09 [22] program packages at the Research Center for Computational Science, Okazaki, Japan. 

## Figures and Tables

**Table 1:** ^13^C and ^1^H-NMR data of compounds **1** and **2** in CDCl_3_.

No.	1				2			
	^13^C		^1^H (*J* in Hz)		^13^C		^1^H (*J* in Hz)	
1	167.0	s	-	-	166.5	s	-	-
2	119.0	s	-	-	128.2	s	-	-
3	106.0	d	-	6.38 brs	140.0	d	-	6.63 brd (6.7)
4	156.4	s	-	-	23.8	t	a	2.34 m
-	-	-	-	-	-	-	b	2.21 ddd (3.1, 6.7, 17.7)
5	38.1	t	-	3.28 s (2H)	81.9	d	-	4.30 dd (3.1, 12.9)
6	130.9	s	-	-	38.9	s	-	-
7	127.2	d	-	5.24 m	31.8	t	α	1.56 m
-	-	-	-	-	-	-	β	1.30 m
8	26.3	t	-	2.16 m (2H)	17.4	t	-	1.51 m (2H)
9	39.2	t	-	2.06 m (2H)	37.8	t	α	0.97 dt (3.1, 12.6)
-	-	-	-	-	-	-	β	1.67 m
10	139.7	s	-	-	38.9	s	-	-
11	124.9	d	-	5.24 m	46.6	d	-	1.73 dd (2.6, 11.3)
12	75.9	d	-	4.56 dt (8.4, 5.9)	17.5	t	α	1.55 m
-	-	-	-	-	-	-	β	1.47 m
13	39.4	t	a	2.58 dd (5.9, 15.0)	41.6	t	α	1.65 m
-	-	-	b	2.23 m	-	-	β	1.70 m
14	139.8	s	-	-	73.0	s	-	-
15	119.2	d	-	5.33 m	58.8	d	-	1.05 brs
16	29.9	t	-	2.16 m (2H)	26.0	t	a	1.68 m
-	-	-	-	-	-	-	b	1.54 m
17	24.6	t	-	2.48 t (7.5) (2H)	28.4	t	a	2.44 m
-	-	-	-	-	-	-	b	2.39 m
18	124.5	s	-	-	125.4	s	-	-
19	110.9	d	-	6.26 brs	110.9	d	-	6.29 brs
20	142.7	d	-	7.34 brs	142.6	d	-	7.35 brt (1.5)
21	138.9	d	-	7.21 brs	138.6	d	-	7.25 brs
22	68.3	t	a	4.39 d (13.0)	30.5	q	-	1.18 s
-	-	-	b	4.20 d (13.0)	-	-	-	-
23	16.7	q	-	1.70 brs	15.9	q	-	1.03 s
24	15.9	q	-	1.60 brs	17.4	q	-	0.87 s
25	147.7	d	-	7.96 brs	16.9	q	-	1.92 s

## Supplementary Materials

The following are available online at www.mdpi.com/1660-3397/15/8/249/s1. Figure S1: ^1^H-NMR spectrum of compound **1**; Figure S2: ^13^C-NMR spectrum of compound **1**; Figure S3: HSQC spectrum of compound **1**; Figure S4: HMBC spectrum of compound **1**; Figure S5: COSY spectrum of comound **1**; Figure S6: ^1^H-NMR spectrum of compound **3**; Figure S7: ^1^H-NMR spectrum of compound **2**; Figure S8: ^13^C-NMR spectrum of compound **2**; Figure S9: HSQC spectrum of compound **2**; Figure S10: HMBC spectrum of compound **2**; Figure S11: COSY spectrum of compound **2**; Figure S12: HSQC-TOCSY spectrum of compound **2**; Figure S13: ECD spectrum of compound **2**; Figure S14: NOESY spectrum of compound **2**.
