# Supplementary material for: Two Furanosesterterpenoids from the Sponge Luffariella variabilis
Source: Mar Drugs. 2017 Aug 10;15(8):249. doi: 10.3390/md15080249 (PMC5577604; doi:10.3390/md15080249)
Supplement: Supplementary file 1 [file marinedrugs-15-00249-s001.pdf]

Two Furanosesterterpenes from the sponge *Luffariella variabilis*

Peni Ahmadi [1], Masahiro Higashi [1], Nicole J. de Voogd [2], Junichi Tanaka [1,*]

[1] *Department of Chemistry, Biology and Marine Science, University of the Ryukyus, Nishihara, Okinawa, 903-0213, Japan.*

[2] *Naturalis Biodiversity Center, P.O. Box 9517, 2300 RA Leiden, the Netherlands.*

Contents

Figure S1. ^{1}H-NMR spectrum of compound **1** 2

Figure S2. ^{13}C-NMR spectrum of compound **1** 2

Figure S3. HSQC spectrum of compound **1** 3

Figure S4. HMBC spectrum of compound **1** 3

Figure S5. COSY spectrum of compound **1** 4

Figure S6. ^{1}H-NMR spectrum of compound **3** 4

Figure S7. ^{1}H-NMR spectrum of compound **2** 5

Figure S8. ^{13}C-NMR spectrum of compound **2** 5

Figure S9. HSQC spectrum of compound **2** 6

Figure S10. HMBC spectrum of compound **2** 6

Figure S11. COSY spectrum of compound **2** 7

Figure S12. HSQC-TOCSY spectrum of compound **2** 7

Figure S13. ECD spectrum of compound **2** 8

Figure S14. NOESY spectrum of compound **2** 8

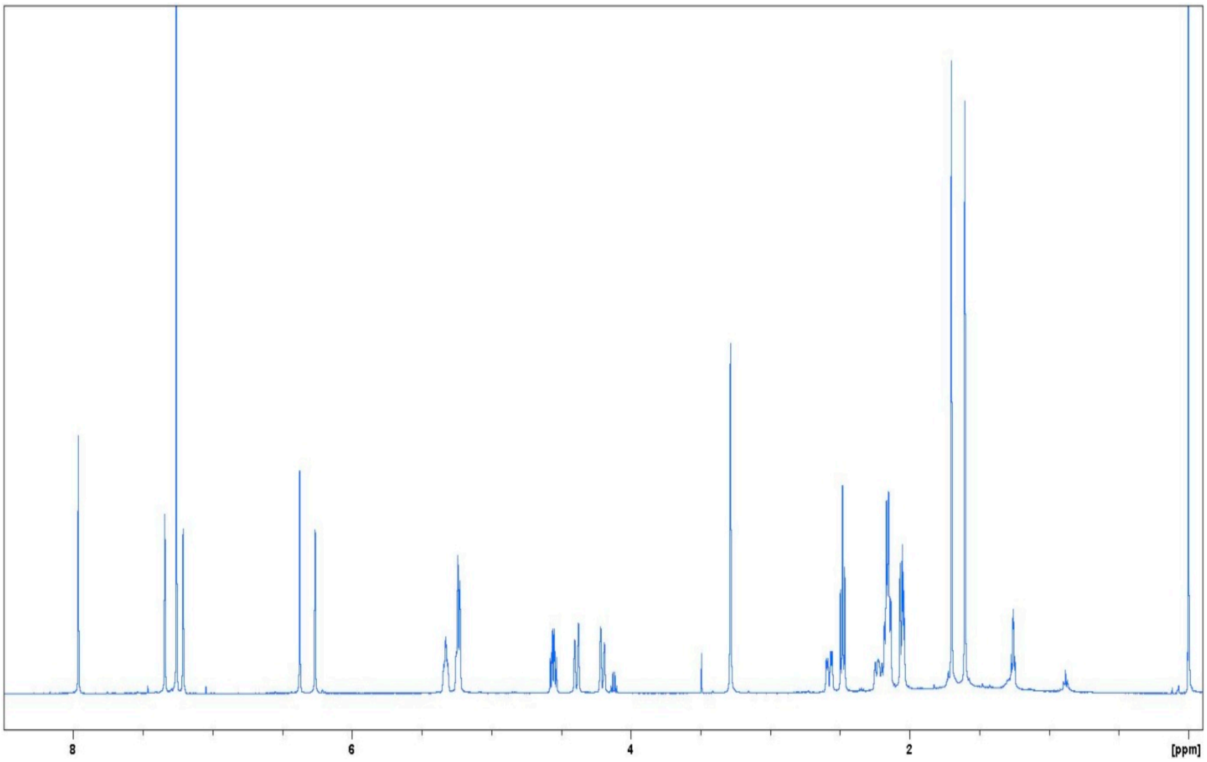

Figure S1. ^{1}H-NMR spectrum of compound **1** ($CDCl_3$, 500 MHz).

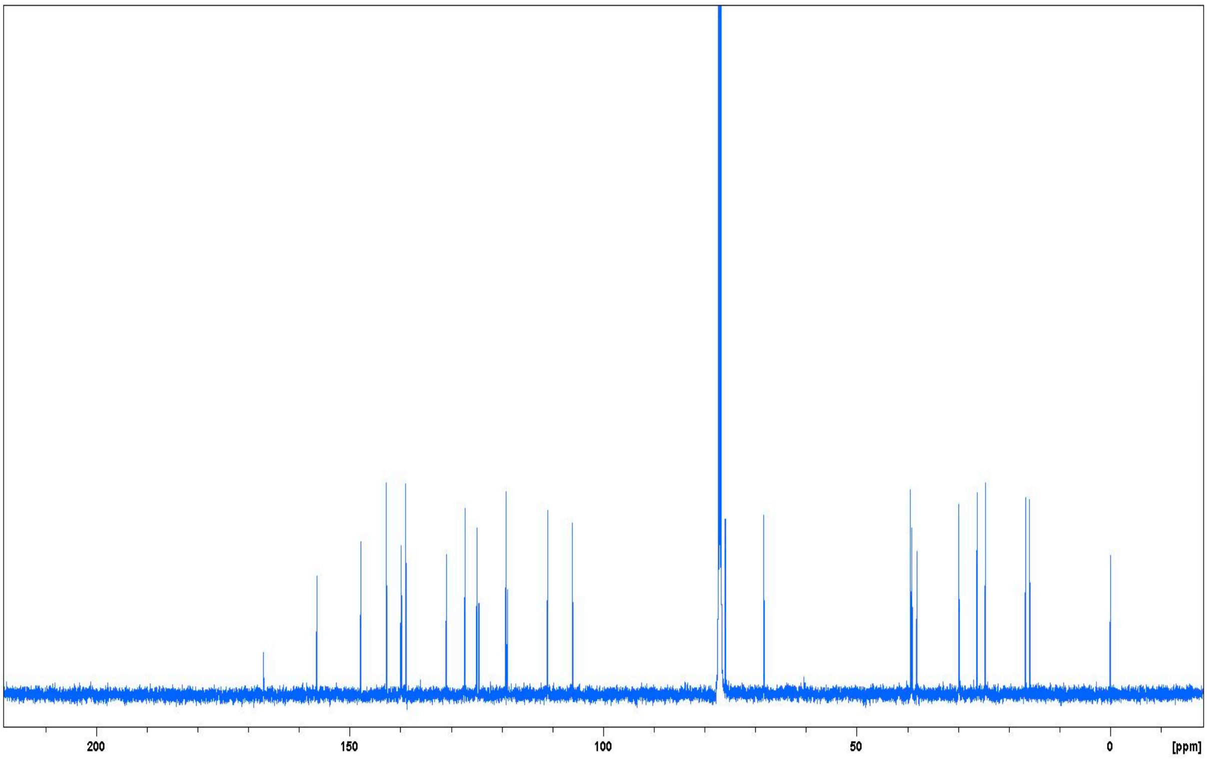

Figure S2. ^{13}C-NMR spectrum of compound **1** ($CDCl_3$, 125 MHz).

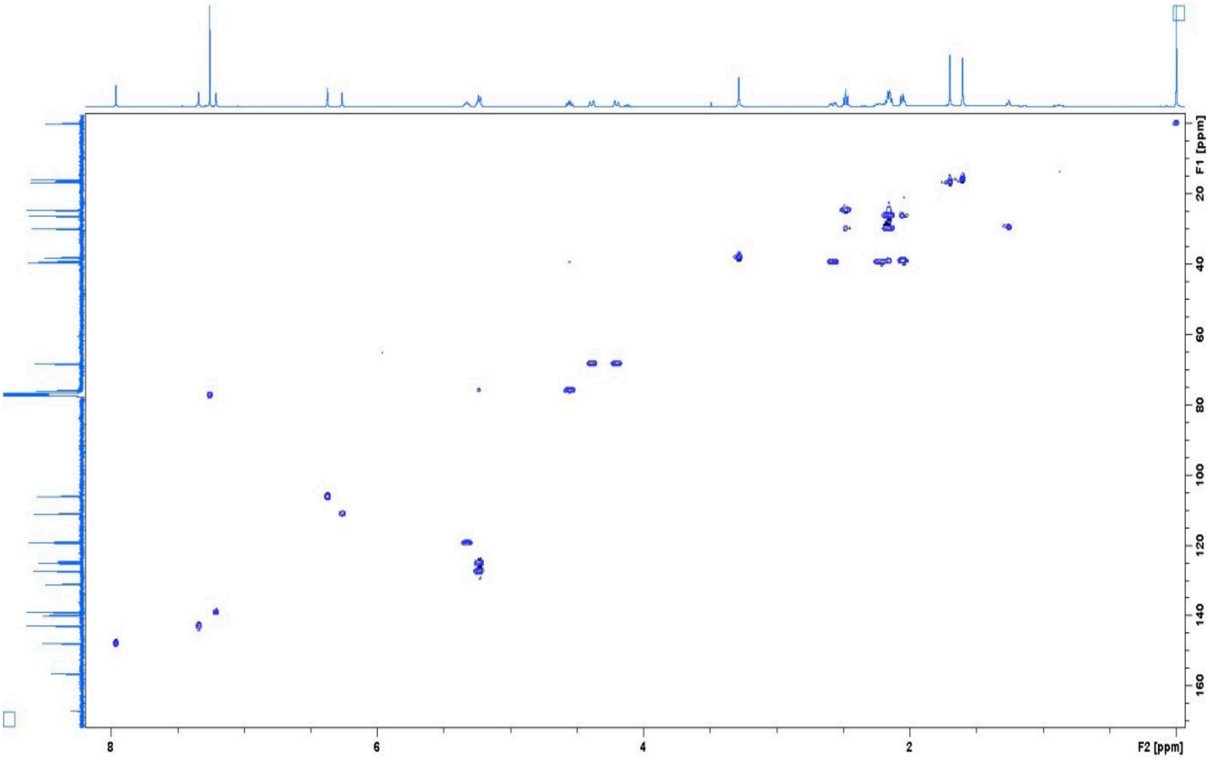

Figure S3. HSQC spectrum of compound **1** ($CDCl_3$, 500 MHz).

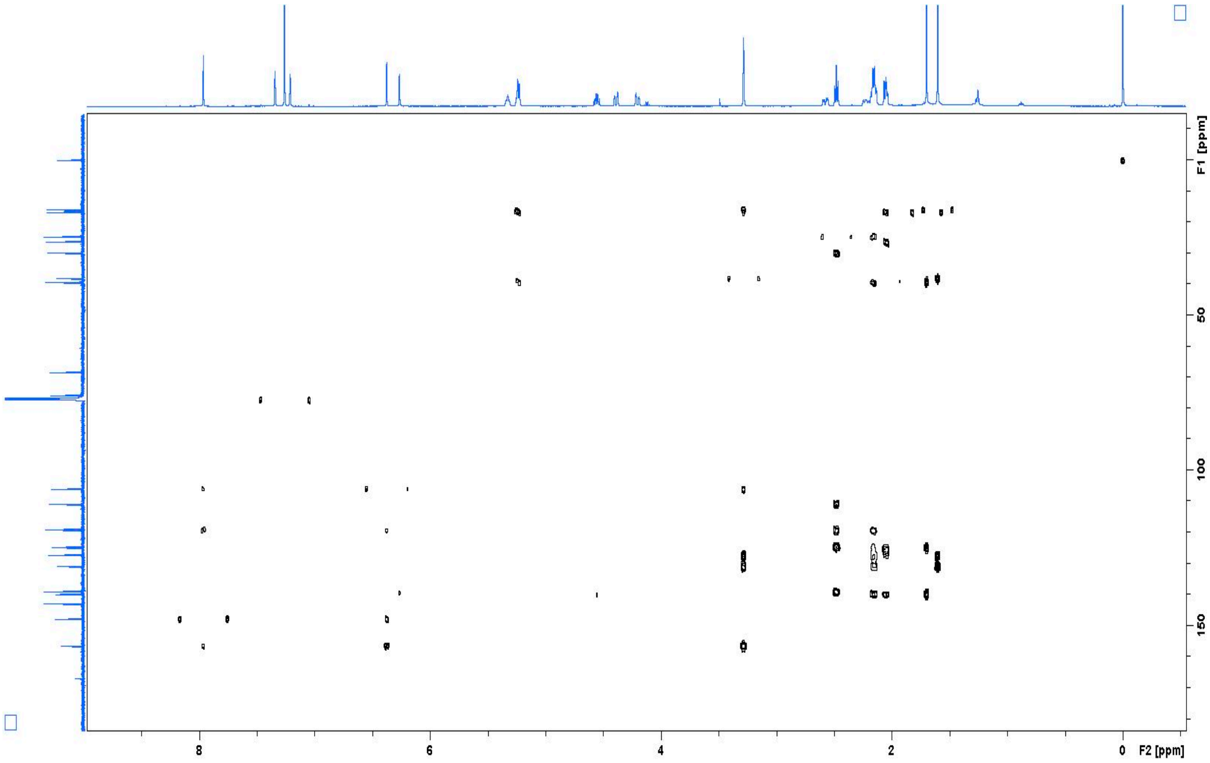

Figure S4. HMBC spectrum of compound **1** ($CDCl_3$, 500 MHz).

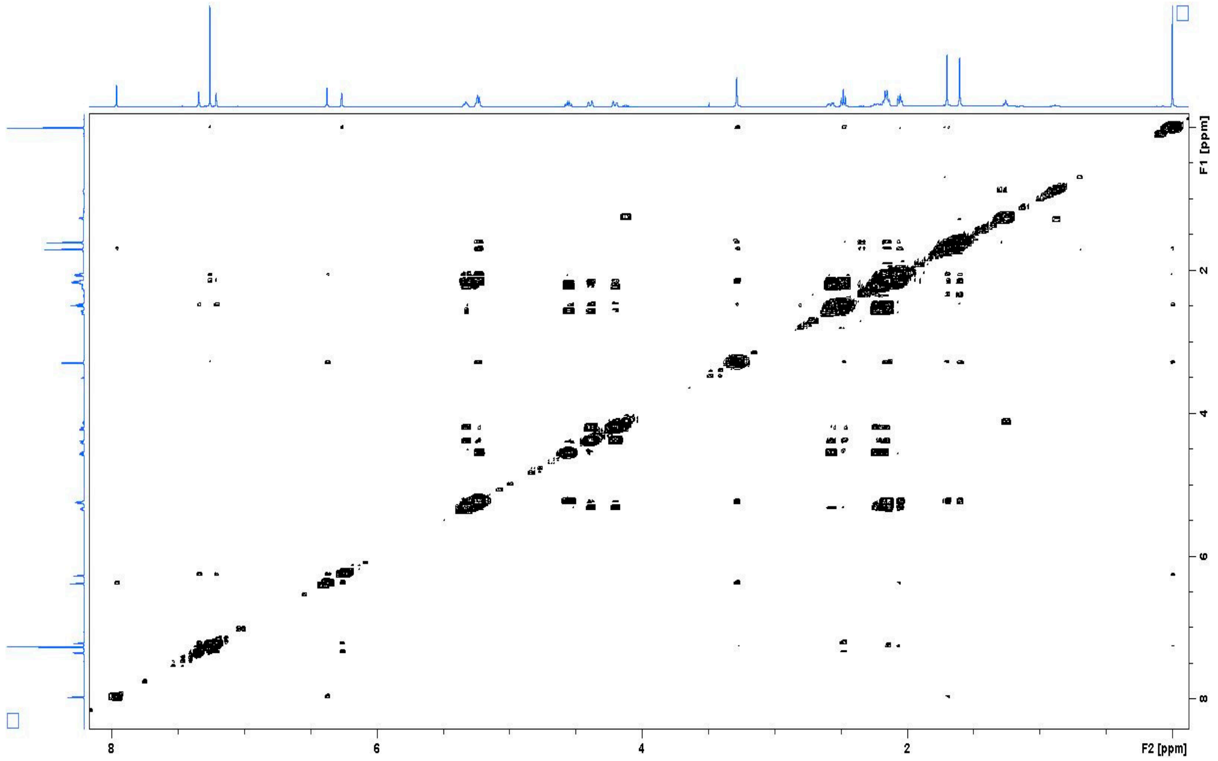

Figure S5. COSY spectrum of compound **1** ($CDCl_3$, 500 MHz).

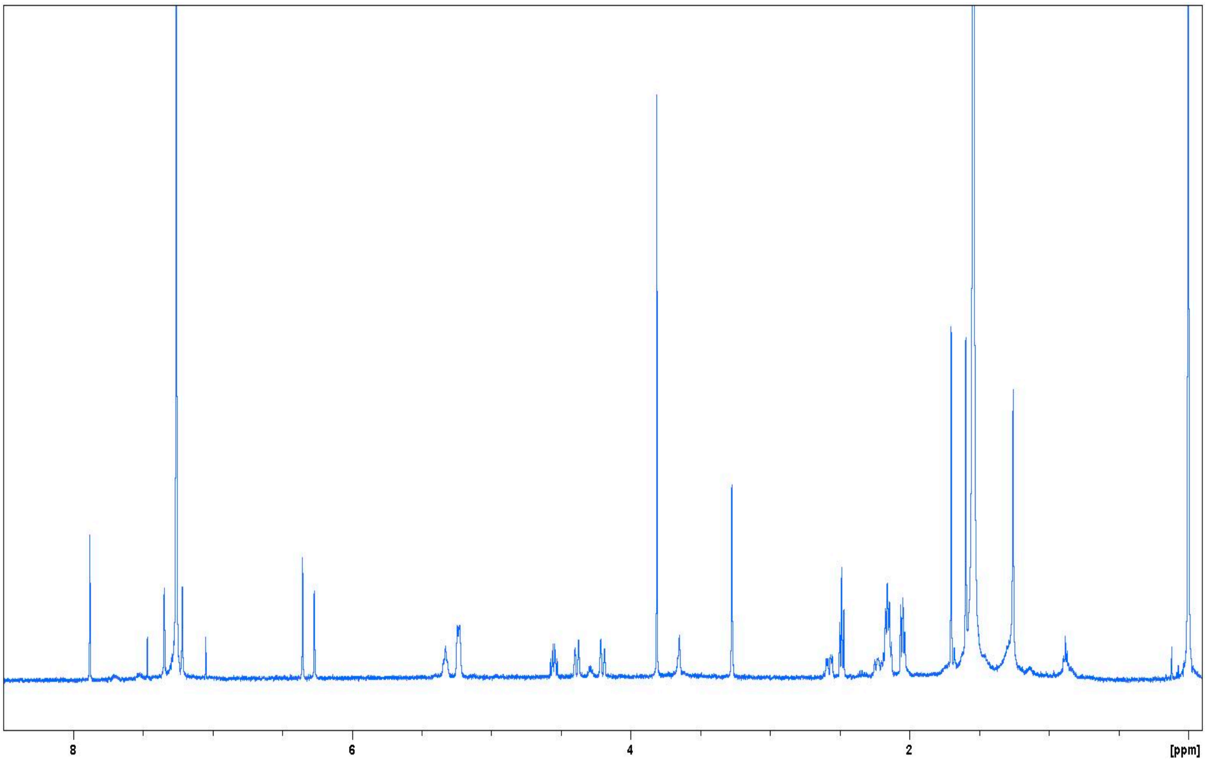

Figure S6. ^{1}H-NMR spectrum of compound **3** ($CDCl_3$, 500 MHz).

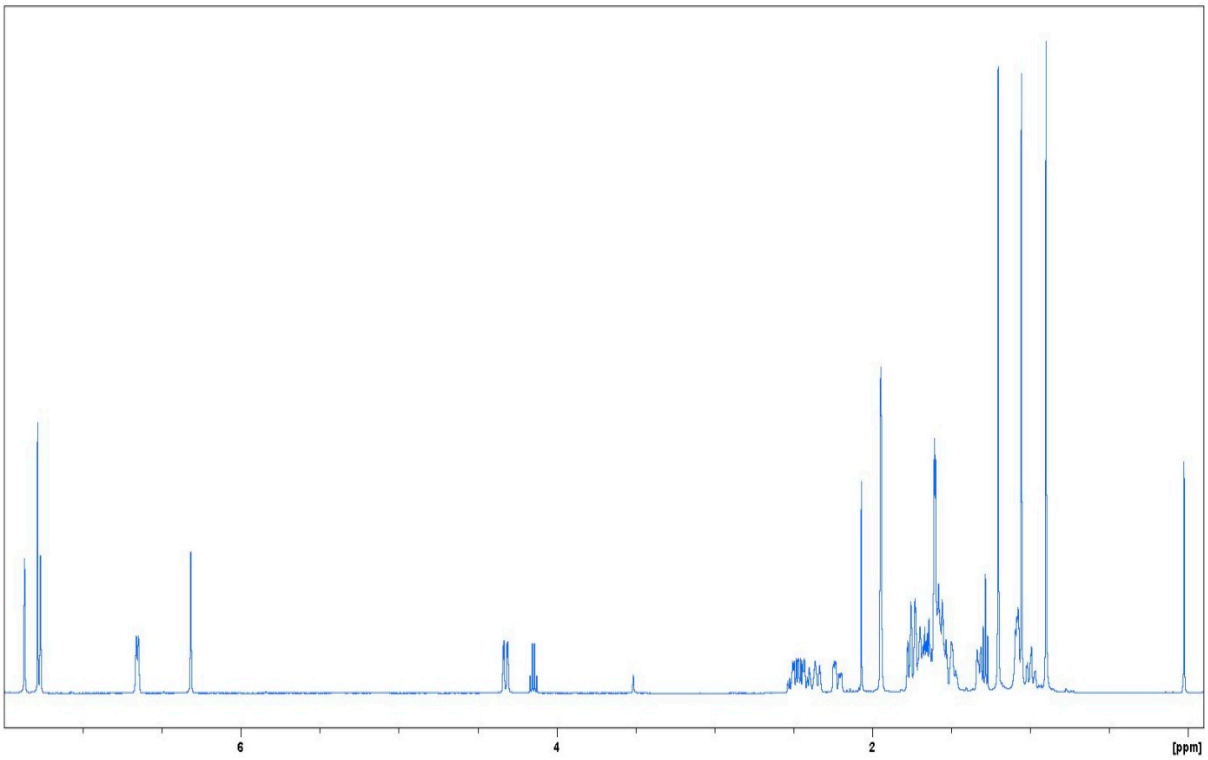

Figure S7. ^{1}H-NMR spectrum of compound **2** ($CDCl_3$, 500 MHz).

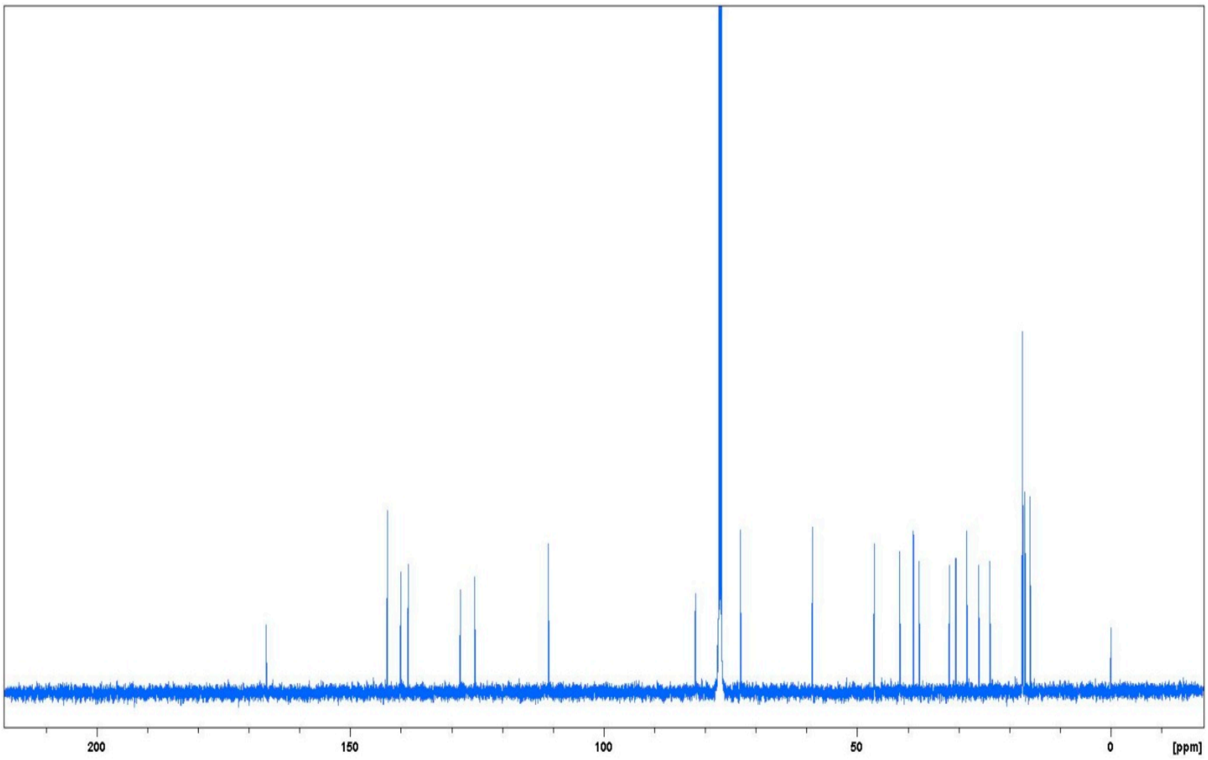

Figure S8. ^{13}C-NMR spectrum of compound **2** ($CDCl_3$, 125 MHz).

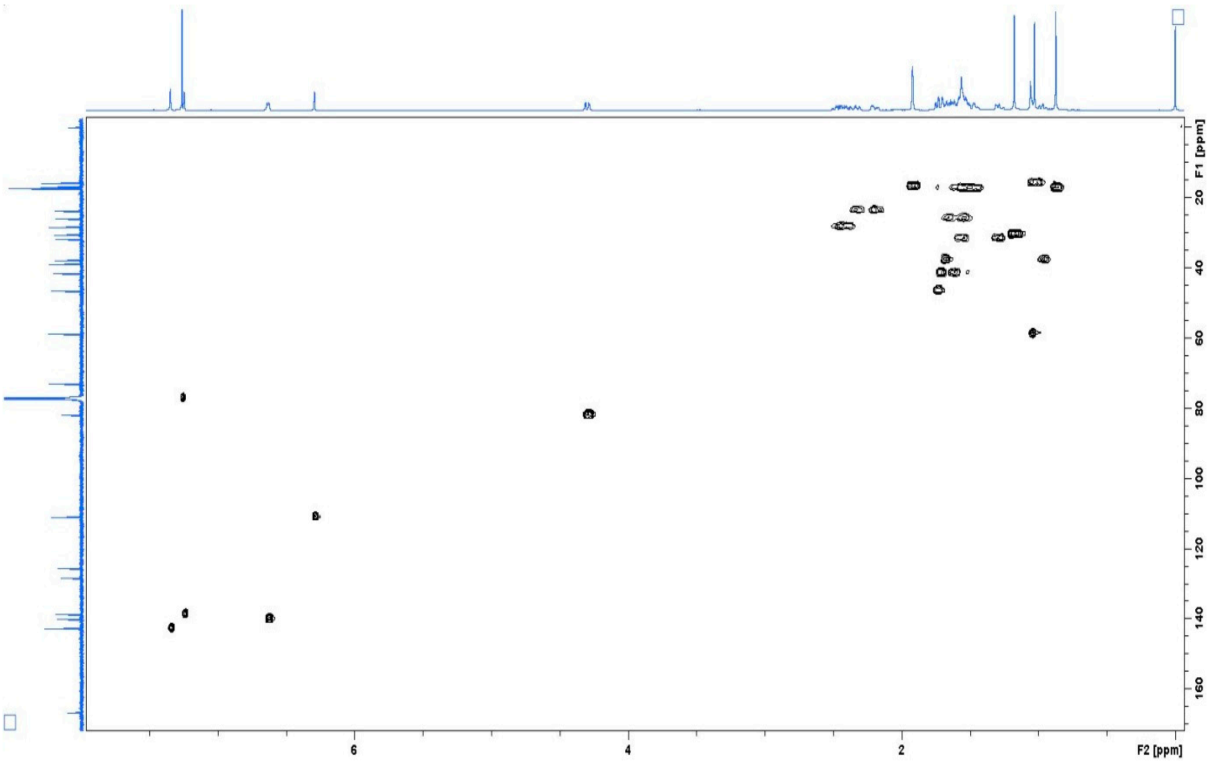

Figure S9. HSQC spectrum of compound **2** ($CDCl_3$, 500 MHz).

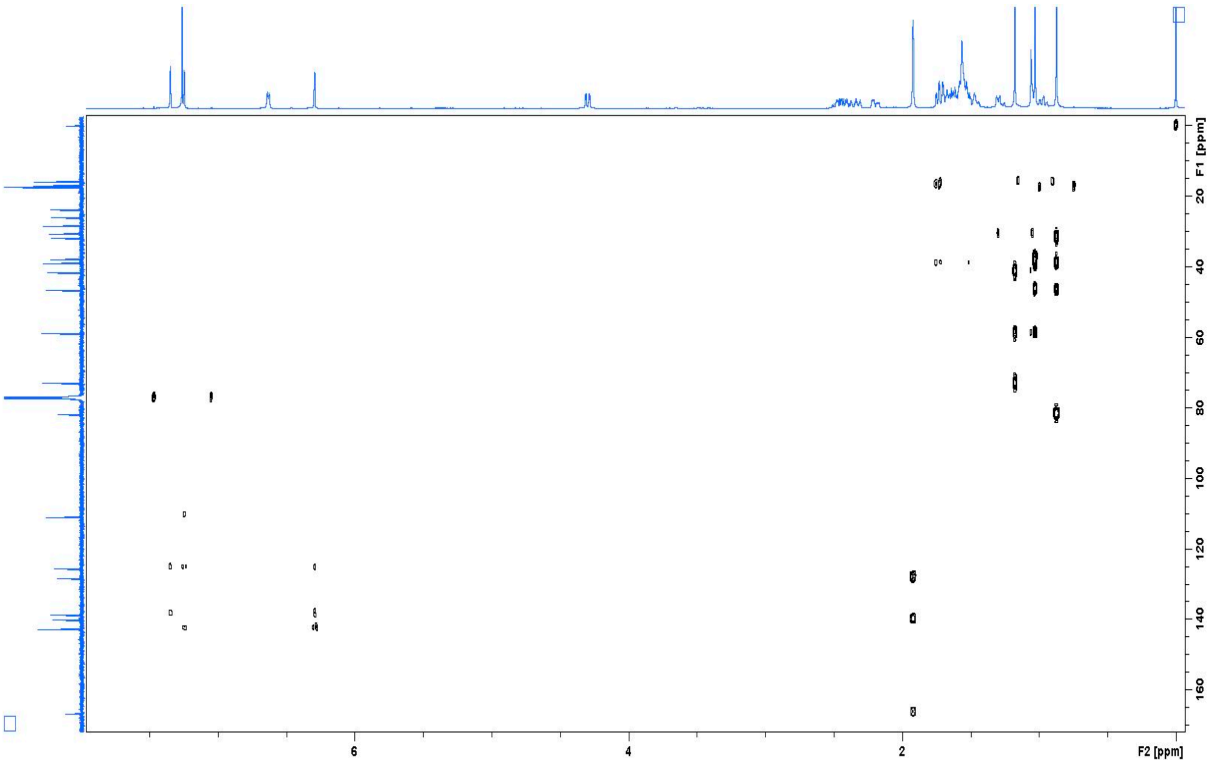

Figure S10. HMBC spectrum of compound **2** ($CDCl_3$, 500 MHz).

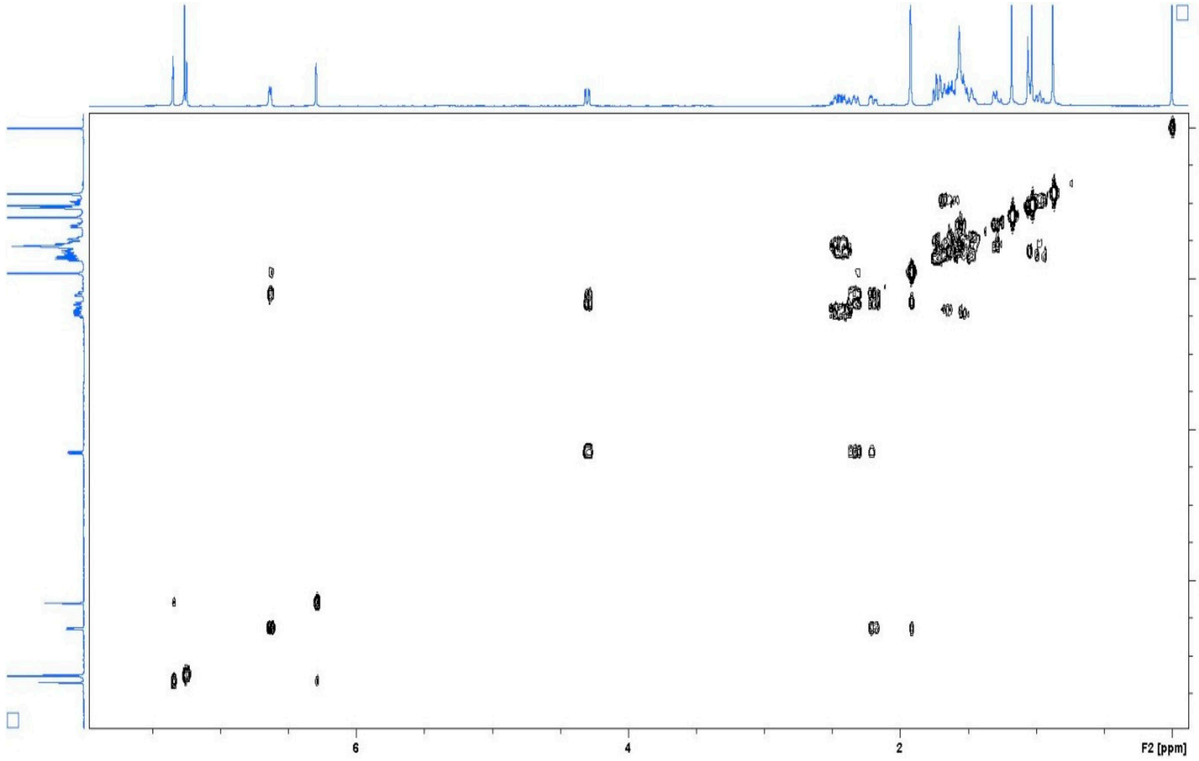

Figure S11. COSYspectrum of compound **2** ($CDCl_3$, 500 MHz).

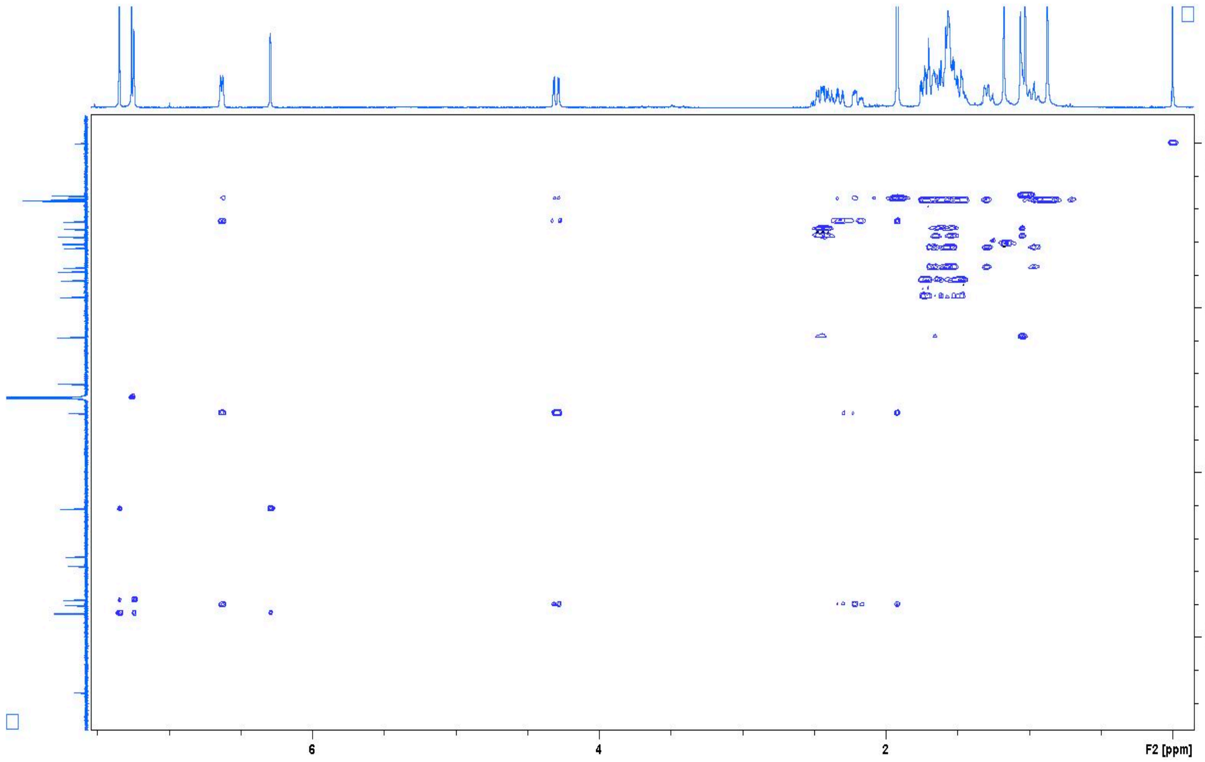

Figure S12. HSQC-TOCSY spectrum of compound **2** ($CDCl_3$, 500 MHz).

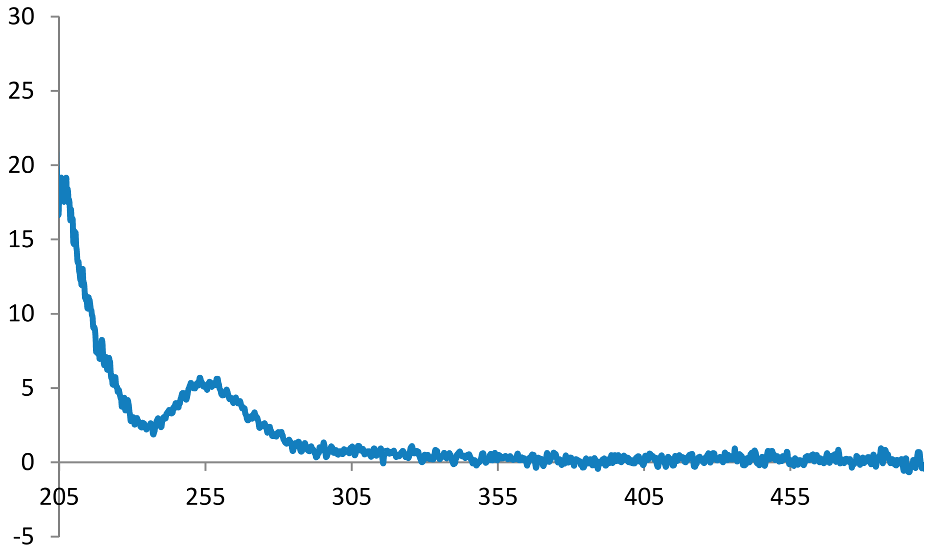

Figure S13. ECD spectrum of compound **2**.

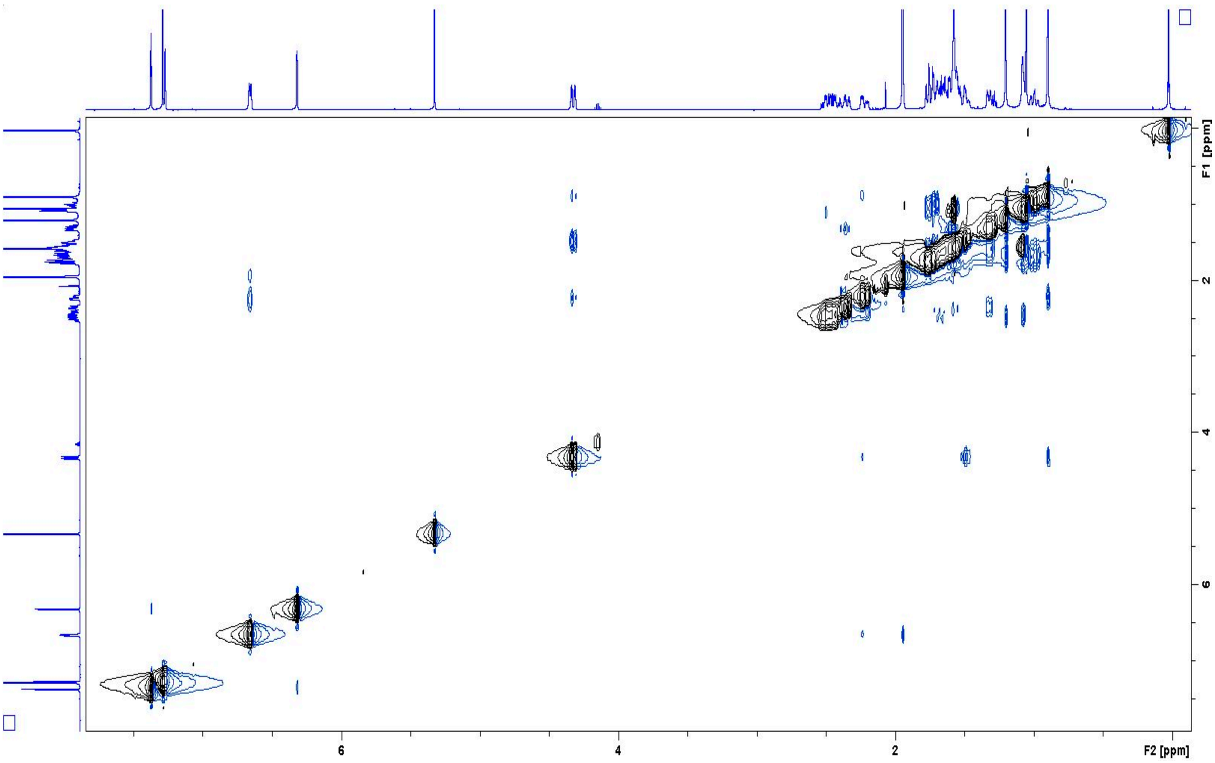

Figure S14. NOESY spectrum of compound **2** ($CDCl_3$, 500 MHz).
